# Molecular analysis of endotracheal tube biofilms and tracheal aspirates in the pediatric intensive care unit

**DOI:** 10.12715/apr.2017.4.14

**Published:** 2017-11-10

**Authors:** Matthew K. Leroue, J. Kirk Harris, Katherine M. Burgess, Mark J. Stevens, Joshua I. Miller, Marci K. Sontag, Yamila L. Sierra, Brandie D. Wagner, Peter M. Mourani

**Affiliations:** 1 Department of Pediatrics, Section of Emergency Medicine, University of Colorado School of Medicine, Anschutz Medical Center, and Children’s Hospital Colorado, Aurora, CO, USA; 2 Department of Pediatrics, Section of Pulmonary Medicine, University of Colorado School of Medicine, Anschutz Medical Center, and Children’s Hospital Colorado, Aurora, CO, USA; 3 Department of Epidemiology, Colorado School of Public Health, Anschutz Medical Center, Colorado School of Public Health, Aurora, CO, USA; 4 Children’s Hospital Colorado, Aurora, CO, USA; 5 Department of Biostatistics and Informatics, Colorado School of Public Health, Aurora, CO, USA; 6 Department of Pediatrics, Section of Critical Care, University of Colorado School of Medicine, Anschutz Medical Center, and Children’s Hospital Colorado, Aurora, CO, USA

## Abstract

**Background:**

Ventilator-associated pneumonia (VAP) is a known complication of mechanically ventilated children in the pediatric intensive care unit (PICU). Endotracheal tube (ETT) biofilms are often implicated in the development of VAP by providing a conduit for pathogens to the lower respiratory tract.

**Methods:**

A prospective cohort study from April 2010–March 2011 of children 4 weeks to 18 years of age ventilated for greater than 72 hours to determine the microbiota of ETT biofilms and tracheal aspirates.

**Results:**

Thirty-three patients were included with a mean age of 6.1 years (SD ± 5.1 years) and average length of intubation of 8.8 days (SD ± 5.0 days). Bacterial communities from tracheal aspirates and the proximal and distal ends of ETTs were determined using 16S rRNA gene libraries. Statistical analysis utilized two-part statistics and the Wilcoxon signed rank sum test for comparison of bacterial communities. Sequencing revealed a predominance of oropharyngeal microbiota including *Prevotella* and *Streptococcus* spp. Pathogenic bacterial genera including *Staphylococcus, Burkholderia, Moraxella,* and *Haemophilus* were also represented. Bacterial load was greatest at the proximal aspect of the ETT. Duration of intubation did not significantly impact bacterial load. Morisita Horn analysis across sites showed similar communities in 24/33 (72%) of patients.

**Conclusions:**

ETT biofilms and tracheal aspirates of intubated patients in the PICU primarily consisted of oropharyngeal microbiota, but had a significant representation of potentially pathogenic genera. While the majority of patients had similar microbiota when comparing their ETT biofilms and tracheal aspirates, a subset of patients showed a divergence between communities that requires further investigation.

## Introduction

Ventilator-associated pneumonia (VAP) is a complication of mechanical ventilation support in critically ill children and is the second most common hospital acquired infection among patients in the pediatric intensive care unit (PICU) [1]. VAP is associated with a substantial increase in resource utility, length of stay, and morbidity [2], yet limited understanding of the microbial factors associated with VAP pathogenesis has precluded development of effective prevention strategies.

It has been postulated that the presence of an endotracheal tube (ETT) contributes to the development of VAP via colonization and formation of biofilms, providing a conduit for potential pathogens to the lower respiratory tract. Multiple studies have indicated that ETTs are quickly colonized with microorganisms and lower airways are exposed to these organisms, increasing risk of VAP or other systemic infection [3]. Biofilms are also relatively protected from the host immune defense and from systemically administered antibiotics [4,5,6]. Further understanding of the composition of microbial communities in ETT biofilms and the timing of colonization could provide insight into mechanisms leading to VAP and creation of preventive interventions.

Tracheal aspirates are often utilized for the diagnosis of VAP in lieu of gold standard methods, such as culture of lower airway samples or lung biopsy, because of the invasiveness of these techniques [7,8,9]. Unfortunately, a recent study by Willison et al. demonstrated that, while tracheal aspirates in the pediatric population are fairly sensitive, they lack specificity and poorly distinguish between infection and colonization, even when stringent requirements for number of colony forming units and polymorphonulcear leukocytes are used to define infection [10]. Furthermore, molecular methods of bacterial identification have demonstrated enhanced detection of pathogenic bacteria compared to traditional culture of bronchoalveolar lavage samples in cystic fibrosis patients [11]. Similar results have been found when using 16S ribosomal RNA (16S rRNA) to analyze central venous catheters [12]. These studies, and others, have called into question the accuracy of traditional methods to identify the most abundant or, potentially, pathogenic bacteria when compared to molecular diagnostics [12,13,14].

There has been limited application of 16S rRNA sequencing to detect bacteria in ETT biofilms and tracheal aspirates. Existing studies, performed in adults, suggest that molecular diagnostics can characterize a larger proportion of the microbial community and provide additional data to better determine whether organisms are more likely to represent infection or colonization compared to traditional approaches. These techniques may also provide insight into the timing of colonization of the lower airways as well as the transition from colonization to infection [9,12,15,16].

The goal of this study was to examine the bacterial composition of ETT biofilms and tracheal aspirates of mechanically ventilated children on the day of extubation to determine whether pathogenic bacteria are disproportionally represented in the ETT biofilm and how the biofilm composition compares to the bacterial communities in the lower airways as characterized by the tracheal aspirate. We hypothesized that the bacterial composition of ETT biofilms and tracheal aspirates are likely similar and will also contain high levels of opportunistic pathogens implicated in lower airway infections.

## Materials and methods

### Data collection

The data and specimens for this analysis were obtained from a prospective study conducted in the Children’s Hospital Colorado PICU between April 2010 and April 2011. The Colorado Multiple Institutional Review Board approved the protocol, and parents or guardian provided informed consent for patients. Children between the age of 4 weeks and 18 years of age who required mechanical ventilator support via ETT for at least 72 hours were eligible for this study. Exclusion criteria included gestational age less than 37 weeks at birth for children less than one year at the time enrollment, indwelling tracheostomy or tracheostomy expected to be placed within seven days of PICU admission, an ETT present without mechanical ventilator support, and contraindication to deep tracheal suctioning.

All patients were subject to the VAP Event Bundle instituted at Children’s Hospital Colorado which includes regular oral care, in-line suctioning, and other infection prevention measures defined in ventilator care guidelines.

Tracheal aspirate specimens from eligible subjects were collected with the first routine suctioning of the ETT occurring on the planned day of extubation via in-line suction and sterile specimen trap. Depth of suctioning was standardized by protocol as 1 cm below the ETT. For samples with low volume, up to 1 mL of sterile saline was used to facilitate collection. Specimens were aseptically transferred from mucous traps to 2 mL cryovials, flash frozen in liquid nitrogen, and stored at −80° C.

ETTs were collected upon extubation of patients, regardless of when extubation occurred. Each ETT was placed in a sterile specimen bag and immediately frozen at −80° C until processed. The proximal 5 cm (defined as the end protruding from the mouth) and the distal 5 cm (defined as the end residing in the trachea) were excised. Two separate standard culture swabs were used to sample the proximal lumen and distal lumen. The ends of the swabs were placed in 400 microliters of QIAGEN Buffer G2 (Germantown, Maryland). The swabs and buffer were then heated to 37° C for 30 minutes and vortexed to help release the biofilm. Afterwards, 200 microliters of the fluid were then used for DNA extraction using QIAGEN EZ1 Advanced DNA Bacterial DNA purification system per the manufacturer’s instructions [17]. The purified DNA was used to determine bacterial load by quantitative PCR (qPCR) [18].

### High-throughput DNA sequencing for microbiome analysis

#### 16S Amplicon Library Construction

Bacterial profiles were determined by broad-range amplification and sequence analysis of 16S rRNA genes as previously described [19,20].

#### Analysis of Illumina Paired-end Reads

Illumina MiSeq paired-end reads were aligned to human reference genome Hg19 with bowtie2 and matching sequences were discarded [21,22]. The remaining non-human paired-end sequences were sorted by sample via barcodes in the paired reads with a python script [20]. Sorted paired-end sequence data were deposited in the NCBI Sequence Read Archive under accession number SRP063527. The sorted paired reads were assembled using phrap, and pairs that did not assemble were discarded [23,24]. Assembled sequence ends were trimmed over a moving window of five nucleotides until average quality met or exceeded 20. Trimmed sequences with more than one ambiguity or shorter than 200 nucleotides were discarded. Potential chimeras identified with Uchime (usearch6.0.203_i86linux32) using the Schloss Silva reference sequences were removed from subsequent analyses [25,26]. Assembled sequences were aligned and classified with SINA (1.2.11) using the 418,497 bacterial sequences in Silva 115NR99 as reference configured to yield the Silva taxonomy [27,28]. Operational taxonomic units were produced by clustering sequences with identical taxonomic assignments. This process generated 6,270,141 sequences for 93 samples (average sequence length: 314 nucleotides; average sample size: 67,421 sequences/sample; minimum sample size: 7,059; maximum samples size: 182,870). The median Goods coverage score was = 99.6% at the rarefaction point of 7,059. The software package Explicet (v2.10.5, www.explicet.org) was used for display and statistical analysis [29,30].

### Statistical analysis

Sequence counts were analyzed using two-part statistics, calculated as the sum of two Chi squared statistics, the McNemar’s test for paired proportions, and the Wilcoxon signed rank sum test as described elsewhere [30]. Morisita Horn (MH) indices were calculated to determine similarity between sample sites. Values range from 0 to 1, with 1 representing complete similarity in the proportion and identity of taxa, and 0 representing no similarity. Wilcoxon signed rank sum tests were performed to analyze differences between each comparison group for both MH and bacterial load. Further bacterial load analysis was done using random coefficients model with random intercept. Additional analyses for sequence (relative abundance of specific taxa), MH, and qPCR were performed using SAS version 9.4, SAS Institute, Cary, NC. Sequence and MH data were calculated in Explicet [31].

## Results

Fifty-seven ETTs were collected. Five ETTs were excluded because the culture swab was unable to pass through the lumen of the ETT (ETT diameter <3.5 mm). Fifty-two ETTs were subject to qPCR amplification. Fifteen ETTs did not have more bacterial DNA than reagent blanks and were eliminated from analysis leaving a total of 37 ETTs for 16S rRNA analysis. Four subjects only had one sample site (proximal or distal ETT or tracheal aspirate) with sufficient biomass to undergo sequencing, leaving 33 patients for comparative analysis. There were 23 paired tracheal aspirate and proximal ETT samples, 30 paired tracheal aspirate and distal ETT samples, and 25 paired distal and proximal ETT samples.

The 33 patients ranged in age from 2 months to 17 years of age. Patients were intubated between 3 days to 22 days with an average duration of intubation of 8.8 days (SD ± 5.0 days). Male patients comprised 58% of the study population. The most common diagnoses were lower respiratory tract infections, seizures, and sepsis. Eight patients received antibiotics 24 hours prior to extubation (Table 1).

### Distribution of bacteria identified

Bacterial communities from the proximal and distal ETT and tracheal aspirates were determined by 16S rRNA analysis. *Prevotella* was the most common genus identified in all sites comprising 36.8% of the overall sequences. *Streptococcus* and *Staphylococcus* were the next most common bacterial genera at 21.6% and 10.2%, respectively (Table 2). All sites contained *Prevotella* and *Streptococcus. Staphylococcus* was absent from two distal biofilms and two tracheal aspirates although not from the same patients. *Staphylococcus, Stenotrophomonas,* and *Veillonella* had a higher abundance in the proximal ETT biofilm compared to the distal ETT biofilm (p<0.01; Table 3). There was a significant difference in the proportion of *Haemophilus* in the tracheal aspirate compared to the proximal ETT (1.51% vs. <1%, p=0.02). *Burkholderia* was more abundant in tracheal aspirate samples (7.35%) versus proximal and distal ETT biofilm samples (1.85% and 1.95%, respectively). However, a significant difference was only observed between the distal ETT and tracheal aspirate (p=0.02, Table 3).

### Bacterial load

qPCR determined bacterial load at each sample site. There was no significant difference between bacterial load of the proximal ETT biofilm and tracheal aspirate. As a group, the sampled tracheal aspirates had a significantly higher bacterial load compared to the distal ETT biofilms (p<0.04). The proximal end of the ETT had greater bacterial load compared the distal end (p<0.01). Random coefficients model with random intercept was used to determine that bacterial load was not significantly associated with length of intubation at any of the sampled sites. There was not a significant interaction between duration of intubation and the comparison of bacterial load across the sampled sites (p=0.35).

### Comparison of diversity

**Figure 1:**
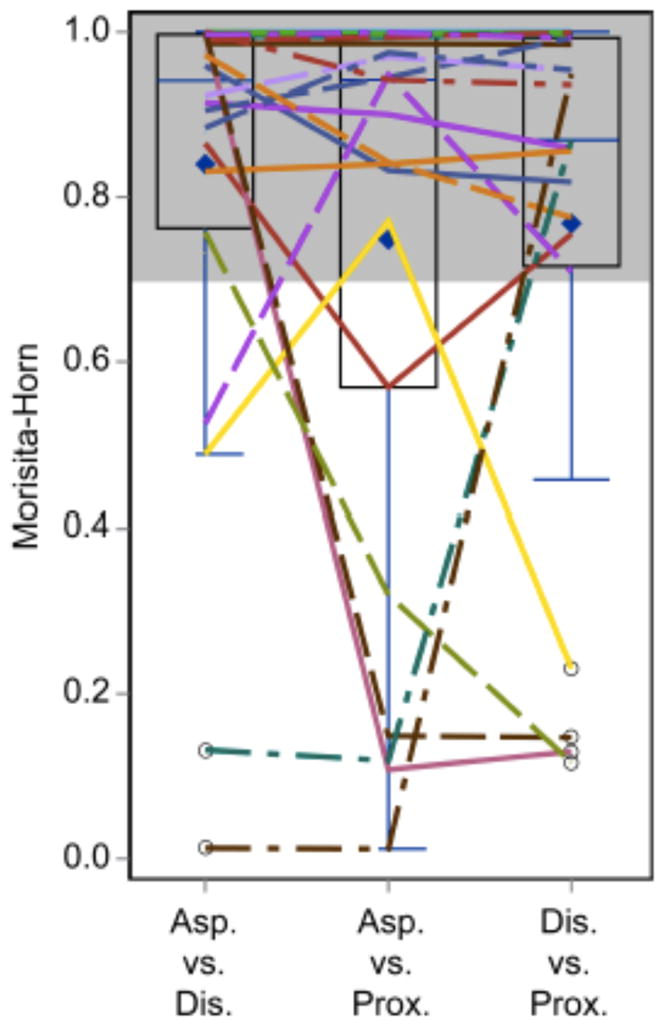
Morisita Horn (MH) analysis comparing bacterial endotracheal tube and tracheal aspirate samples revealed that most sites were similar in their microbial composition (values greater than 0.7). There were nine patients, however, with MH values <0.7 when comparing sites. These patients and the sites responsible for the dissimilarity are represented by the colored lines crossing into the white area. These patients are detailed in Figure 2. Asp: aspirate; Dis: distal; Prox: proximal.

**Figure 2:**
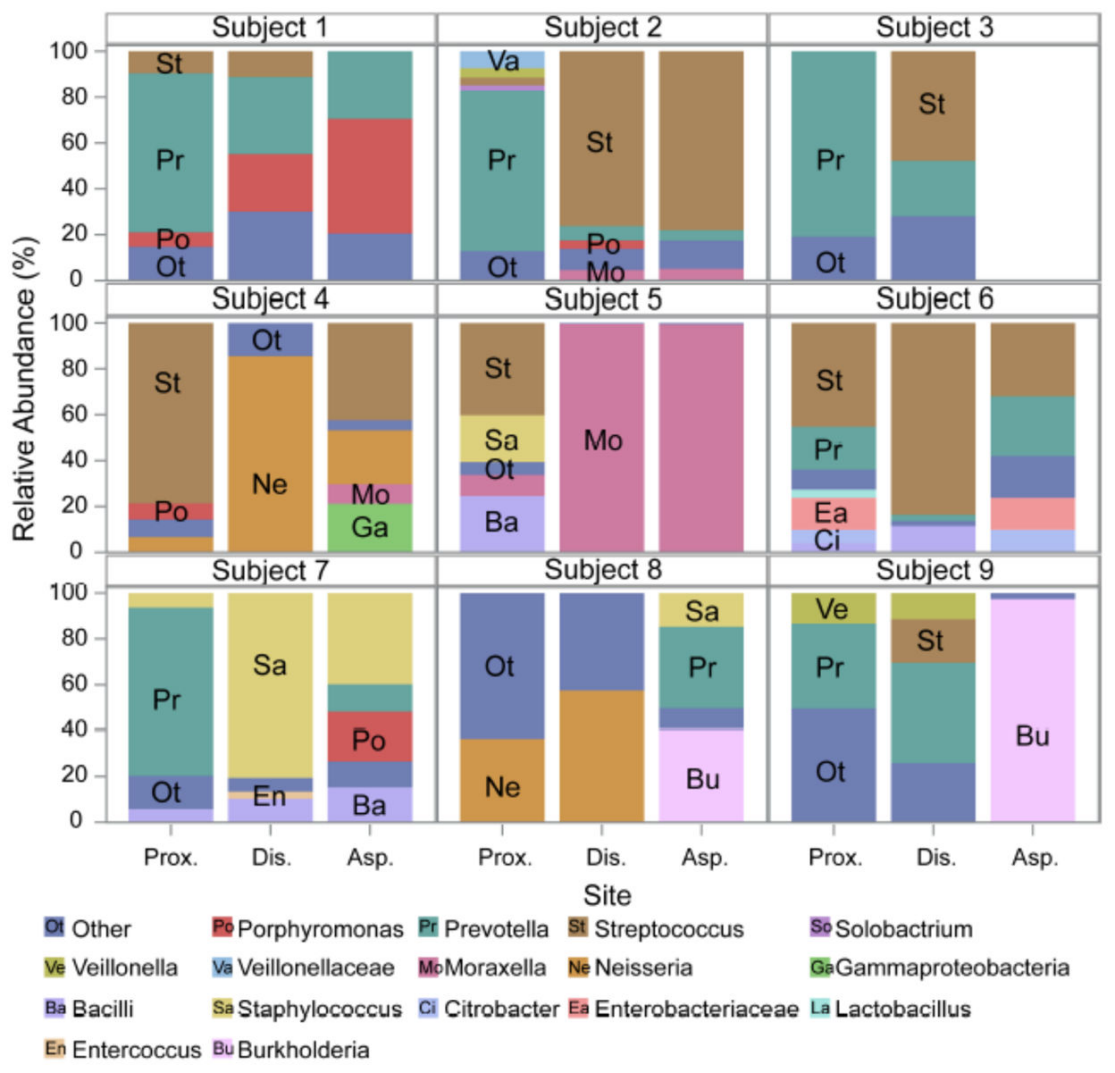
Patients with Morisita Horn less than 0.7 were not consistent in which sampling site was responsible for the dissimilarity. Different genera were responsible for increased diversity in most patients. No tracheal aspirate was available for analysis for Patient 3. Asp: aspirate; Dis: distal; Prox: proximal

The median index for MH comparison was 0.94 between tracheal aspirates with proximal ETT biofilms, 0.94 between tracheal aspirates and distal ETT biofilms, and 0.87 between the distal ETT biofilms and proximal ETT biofilms (Figure 1). While a majority of patients had relative similarity between the sample sites, there was a subset of patients (n=9, 27.3%) with MH index scores less than 0.7, indicating less similarity. These patients were not consistent in which sampling site was responsible for the dissimilarity. There were also various predominant genera in these dissimilar communities that were not consistent between patients (Figure 2). We examined the clinical and demographic data for the subset of patients with a MH index score less than 0.7 in attempt to determine factors that contribute to divergent bacterial populations. There was no significant difference in type of admission diagnosis, age, length of intubation, or total bacterial load between these 9 patients and the remaining cohort. Of the nine patients with MH <0.7, only three patients were on antibiotics 24 hours prior to extubation.

## Discussion

We examined the bacterial composition of ETT biofilms and tracheal aspirates collected on the day of extubation from mechanically ventilated children to determine the relationship between biofilms and lower airway colonization. We found that molecular analysis techniques reveal a wide variety of microbial taxa, well beyond that of standard culture techniques, and may have implications for future strategies to prevent ventilator-associate infections.

This study compared the microbiology of lower airway secretions to that of the ETT biofilm and provides new insight into the complex composition of ETT biofilms and airway colonization which could impact the risk of ventilator-associated infections. 16S rRNA sequencing demonstrated that *Prevotella* was the most prevalent genus in this pediatric population. *Prevotella* is a known constituent of the oropharyngeal microbiota and likely represents a common inoculum within the ETT through contamination with oral secretions either during the process of intubation or via aspiration of oral secretions after intubation has occurred. *Streptococcus* and *Staphylococcus,* other common constituents of the oropharyngeal microbiota, were also prominent in the ETT biofilms and tracheal aspirates. Yet, all three sample sites contain taxa with species that have pathogenic potential [32]. The predominance of *Staphylococcus* in the proximal section of the ETT was an interesting finding and may be a product of external manipulation of the ETT and subsequent introduction of bacteria to the endotracheal circuit. In addition, the proximal aspect of the ETT is least likely to be affected by immune response or antibiotics [33].

*Burkholderia, Neisseria*, *Moraxella,* and *Haemophilus* sequences are potential pathogens that were identified in proximal and distal biofilms as well as tracheal aspirates. While not all of these genera demonstrated a statistically significant difference between sample sites, they were represented in a higher abundance in the distal ETT biofilms and tracheal aspirates. This is not unexpected as these genera are often thought to be potential infectious pathogens in the lower airways [9]. While many investigations of VAP discuss the risk of oral microbiota being introduced to the lower airways through tracheal intubation, it is also evident that the microbiota of the lower airways impact the distal biofilms as well [9,16]. The median MH value on site comparison ranged from 0.87 to 0.94 indicating that the bacterial communities of the tracheal aspirate and distal and proximal biofilms were consistent with each other. There were, however, nine patients with MH values less than 0.7 for one or more comparison between sampling sites. Subsequent analyses did not provide an explanation for the divergence observed in this subset of patients.

Patients with MH index less than 0.7 were neither more likely to be on antibiotics prior to extubation, nor were they noted to have a greater bacterial load at sampled sites than the rest of the cohort. While possible contamination could explain some of the differences observed, that fact that differences were seen within the ETT itself suggests there may be alternative explanations as well. These patients are interesting outliers and further investigation is required to identify factors that contribute to this diversity and its clinical impact on patients.

It has been suggested that strategies to reduce ETT biofilm accumulation may decrease the risk of VAP [3]. However, total bacterial load was found to be significantly higher in the tracheal aspirate compared to the distal ETT. This finding may suggest that the ETT biofilm is a reservoir for infection whereas bacterial replication and biomass is more robust in the airways where nutritive resources are more abundant. Furthermore, there was increased bacterial load at the proximal biofilm when compared to the distal biofilm among patients. This is in contrast to previous data that has reported higher bacterial load in the distal ETT biofilms [34,35,36]. This discrepancy may represent the effect of exposure to the external environment on each section of the ETT or the fact that the proximal ETT is protected from delivery of antibiotics or host immune responses. The duration of intubation did not appear to affect bacterial load at any of the three sample sites, which is consistent with at least one other study [15]. Given that biofilms and tracheal aspirate samples were obtained on the day of extubation, it is difficult to determine the effect of time on bacterial load for each individual patient. However, increased length of intubation as an independent variable does not appear to result in increased bacterial load when assessed in subjects intubated greater than 72 hours. It should be noted that ETTs analyzed just 72 hours after intubation had high levels of bacteria present, demonstrating how quickly bacterial biofilms are formed.

Our data showed that *Streptococcus* was the second most prominent genus in this patient population. *Streptococcus* has been described as forming biofilms especially in conjunction with other bacteria including *Actinomyces* species and *Veillonella* species [37,38]. *Veillonella* species were found in both proximal and distal biofilms whereas *Actinomyces* species were not prominent in our study. Previous studies have isolated *Enterobacteriaceae* species within ETT biofilms though these species were not highly represented in this study [39,40]. The internal lumen of the ETT was sampled in this study which is in contrast to similar studies which have sampled the external and internal aspects of the ETT [15,16]. The internal lumen was selected as it was thought to be least impacted by the host immune response and systemic antimicrobial agents and also less likely to be affected by communication with the external environment with exposure to the bidirectional movement of ventilated gas.

This study does have several limitations. While this study is relatively large compared to similar published work performed utilizing 16S rRNA sequencing of ETT, the study size is still insufficient to make generalized conclusions within this patient population. Fifteen of the ETT did not have more bacterial DNA compared to reagent blanks after amplification with qPCR. Since neither the proximal nor distal portions amplified, the assumption was made that there was not significant biomass in the ETT biofilms. Given that a majority of samples did produce sufficient biomass, it is unlikely that sampling technique contributed to the lack of amplification. It may have been possible to increase yield of sampling using flocked swabs instead of standard culture swabs. Another limitation of the methodology employed for bacterial molecular identification is the inability to accurately identify organisms at the species level. Thus, further investigations are required to determine whether genera like *Staphylococcus* and *Streptococcus* are comprised of mostly commensal species versus potentially pathogenic ones. In addition, the 16S rRNA methodology does not detect fungi or viruses which may impact bacterial load and composition as well as host immune responses. Furthermore, there are multiple patient variables that could theoretically impact the microbiome of the subjects including the total use of antibiotics, nasal versus oral placement of ETT, or presence of a cuff. An exhaustive assessment of these variables was beyond the scope of this initial study, although several of the most impactful interventions such as antibiotics within 24 hours of extubation were evaluated. While data such as infection status on admission and antibiotic exposure were analyzed in this study, other factors such as suctioning frequency and consistency of technique were not examined, although each patient was managed per a standardized VAP bundle. Each of these elements could introduce variability in the quantitative assessment of bacterial communities, and a more exhaustive analysis of these factors may be warranted. Investigations evaluating the entire spectrum of microbiota and host responses are likely to produce further insights into lower airway colonization and the risks for transition from colonization to infection in mechanically ventilated children.

## Conclusions

We found that there is a broad microbiota in tracheal aspirates and ETT biofilms from intubated pediatric patients. Oropharyngeal bacteria, which are a main source of inoculation of the airways, were most highly represented. However, there were significant numbers of potentially pathogenic genera present in variable abundances demonstrating the complex interaction between the upper and lower airways and the ETT. While a majority of sample sites showed similar microbial communities within patients there were differences between sample sites in nine patients that was not related to age, admission diagnosis, total bacterial load, or antibiotic administration 24 hours prior to extubation. Further investigations are warranted to determine the cause and clinical impact of such differences.

## Figures and Tables

**Table 1:** Patient characteristics (n=33)

	Range	Mean (SD)
Age (years)	0.2–17 years	6.1 (±5.1)
Height (cm)	55–167 cm	109.5 (±30.1)
Weight (kg)	4.1–100 kg	24.8 (±20.0)
Days intubated	3.0–22.0 days	8.8 (±5.0)
PICU length of stay	4.0–41.0 days	13.6 (±8.6)
Hospital length of stay	5.9–143.9 days	30.6 (±26.9)

	Number of patients	Percent

Gender		
Male	19	57.6
Admission category		
Medical	24	72.7
Surgical	1	3.0
Trauma	8	24.3
Admission medical diagnosis		
Lower respiratory tract infection	4	16.7
Seizures	6	25.0
Sepsis	3	12.5
Non-infectious airway obstruction	3	12.5
Other	8	33.3
Received antibiotics 24 hours prior to extubation	8	24.2

**Table 2:** Mean relative abundance of bacteria in sampled sites

Bacteria	Overall	Proximal	Distal	Tracheal Aspirate
*Prevotella*	36.78%	41.09%	35.54%	35.22%
*Streptococcus*	21.55%	16.64%	24.57%	21.69%
*Staphylococcus*	10.21%	16.77%	7.60%	8.59%
*Burkholderia*	3.94%	1.85%	1.95%	7.35%
*Moraxella*	3.92%	<1%	6.33%	3.78%
*Porphyromonas*	2.79%	1.51%	2.41%	4.01%
*Neisseria*	2.70%	1.46%	3.15%	3.04%
*Bacilli*	1.99%	2.45%	3%	<1%
*Veillonella*	1.80%	2.51%	1.81%	<1%
*Stenotrophomonas*	1.34%	<1%	<1%	2.38%
*Gammaproteobacteria*	<1%	<1%	<1%	1.82%
*Haemophilus*	<1%	<1%	1.41%	1.51%
*Lacobacillales*	<1%	1.48%	<1%	<1%
*Carnobacteriaceae*	<1%	1.45%	<1%	<1%
Other	12.98%	12.79%	12.25%	10.61%

**Table 3:** Comparison of bacterial genera across sampled sites

Bacteria	Distal vs Proximal			Aspirate vs Proximal			Aspirate vs Distal		
	Number disagree	Median relative abundance*	P-value	Number disagree	Median relative abundance^†^	P- value	Number disagree	Median relative abundance^‡^	P-value
Acinetobacter	7 (0.28)	0.002	0.39	9 (0.39)	−0.006	0.11	11 (0.35)	0.001	0.45
Burkholderia	8 (0.32)	−0.001	0.81	9 (0.39)	−0.005	0.07	10 (0.32)	−0.005	0.02*
Haemophilus	6 (0.24)	0.006	0.37	5 (0.22)	−0.029	0.02*	6 (0.19)	−0.002	0.91
Moraxella	10 (0.40)	−0.002	0.29	7 (0.30)	−0.028	0.06	15 (0.48)	−0.010	0.58
Neisseria	0 (0.00)	0.011	0.29	3 (0.13)	0.033	0.08	3 (0.10)	0.001	0.47
Porphyromonas	4 (0.16)	0.002	0.32	1 (0.04)	0.076	0.97	6 (0.19)	−0.008	0.26
Prevotella	0 (0.00)	−0.063	0.98	0 (0.00)	1.929	0.54	0 (0.00)	0.221	0.55
Staphylococcus	0 (0.00)	1.854	<0.01*	0 (0.00)	0.052	0.99	0 (0.00)	−0.040	0.16
Stenotrophomonas	11 (0.44)	0.007	<0.01*	9 (0.39)	0.004	0.53	13 (0.42)	−0.001	0.08
Streptococcus	0 (0.00)	−0.003	0.74	0 (0.00)	1.463	0.45	0 (0.00)	0.016	0.44
Veillonella	0 (0.00)	0.566	<0.01*	0 (0.00)	0.530	0.08	2 (0.06)	0.009	0.78

* p<0.05 Negative values represent a higher relative abundance observed in the Distal site compared to the Proximal site Negative values represent a higher relative abundance observed in the Aspirate site compared to the Proximal site Negative values represent a higher relative abundance observed in the Aspirate site compared to the Distal site
